# Genetic Insights Into Type 2 Diabetes Mellitus Susceptibility: A Case-Control Study of the *ADIPOQ* rs1501299 Polymorphism in the Population of Noakhali Region of Bangladesh

**DOI:** 10.1155/genr/8818420

**Published:** 2025-05-24

**Authors:** Md. Anamul Haque, Md. Sad Salabi Sawrav, Shipan Das Gupta, Shuvo Chandra Das, Dhirendra Nath Barman, Mohammed Mafizul Islam, Md. Murad Hossain

**Affiliations:** Department of Biotechnology and Genetic Engineering, Noakhali Science and Technology University, Noakhali 3814, Bangladesh

**Keywords:** *ADIPOQ* gene, cardiovascular disease (CVD), Hardy–Weinberg equilibrium, PCR-RFLP, rs1501299 polymorphism, type 2 diabetes mellitus (T2DM)

## Abstract

Type 2 diabetes mellitus (T2DM) is a global health concern, particularly prevalent in low to middle-income countries like Bangladesh. This case-control study aims to explore the correlation between the *ADIPOQ* rs1501299 polymorphism and susceptibility to T2DM among the population of Noakhali region of Bangladesh. The study, involving 152 T2DM patients and 118 healthy controls, explores the genetic underpinnings of T2DM, considering the rising prevalence in Bangladesh. The *ADIPOQ* gene, implicated in diabetes development, is examined for the rs1501299 polymorphism, known for its associations with insulin resistance and T2DM in various populations. Genotyping, conducted through PCR and RFLP analysis, reveals significant deviations from Hardy–Weinberg equilibrium for the TT genotype, suggesting potential demographic influences. Clinical and biochemical characteristics, including blood pressure and lipid levels, highlight the complex interplay between genetics, metabolic outcomes and cardiovascular health in T2DM patients. This study identifies a significant association between the *ADIPOQ* rs1501299 T allele and increased T2DM risk, emphasizing the need for personalized risk assessment. However, *ADIPOQ* rs1501299 did not show any substantial association with CVD in the studied population. Despite limitations in sample size and regional focus, this study provides valuable insights into the genetic landscape of T2DM in the Noakhali population, paving the way for future research and personalized therapeutic interventions in addressing the global T2DM epidemic.

## 1. Introduction

Type 2 diabetes mellitus (T2DM) represents a widespread, persistent health concern on a global scale. This condition primarily arises from a combination of genetic predisposition, inadequate or inefficient production and release of insulin by the pancreas, reduced responsiveness of peripheral tissues to insulin and various environmental factors [1–3]. The global prevalence of T2DM has shown a significant upward trend, particularly in low to middle-income countries. In 2017, the number of individuals diagnosed with diabetes worldwide reached 425 million, with an alarming 69 million of those cases being from Bangladesh. This accounted for a prevalence rate of 6.9% and what's noteworthy is that a staggering 90% of the Bangladeshi patients were affected by T2DM [4, 5]. According to the International Diabetes Federation (IDF) in 2021, approximately 536.6 million individuals worldwide, constituting 10.5% of the global population, were afflicted with T2DM. Projections from IDF indicate that by the year 2045, the prevalence of T2DM is anticipated to escalate to 783.2 million individuals, encompassing 12.2% of the global population [6]. In Asia, particularly within the Indian subcontinent, including Bangladesh and China, there has been a distressing surge in an epidemic, with the incidence rate escalating rapidly [7–9]. In rural regions of Bangladesh, a substantial portion of the adult population, ranging from 20% to 30%, exhibits abnormalities in fasting blood sugar levels or impaired glucose tolerance. Projections indicate that by the year 2030, the prevalence of diabetes, primarily T2DM, is expected to surge, with estimates ranging from 24% to 34% [10]. The risk of developing T2DM is influenced by a mix of unmodifiable factors such as age, a family history of diabetes, and one's racial or ethnic background, as well as modifiable factors including obesity, tobacco use, sedentary way of life, dietary habits, and exposure to physical and emotional stress. These risk determinants are closely associated with the rapid urbanization and evolving lifestyle patterns, underscoring the intricate interplay between both genetic and environmental influences on the disease's onset and progression [11–16]. T2DM poses a substantial challenge to the public healthcare system due to its propensity to give rise to a spectrum of complications that can adversely impact various vital organs, including the heart, kidneys and eyes. Recent advances in the field of genetics have leveraged Genome-Wide Association Studies (GWAS), leading to the discovery of over 100 genomic loci associated with susceptibility to T2DM. These studies have also pinpointed distinct genetic markers that exhibit significant correlations with conditions such as obesity, diabetes and cardiovascular disease (CVD) [17–21]. Furthermore, numerous research investigations have demonstrated that a variety of genes participate in the regulation of glucose metabolism, beta-cell performance and insulin secretion pathways, both individually and in concert [22]. This intricate interplay ultimately leads to the development of T2DM.

**Figure 1:**
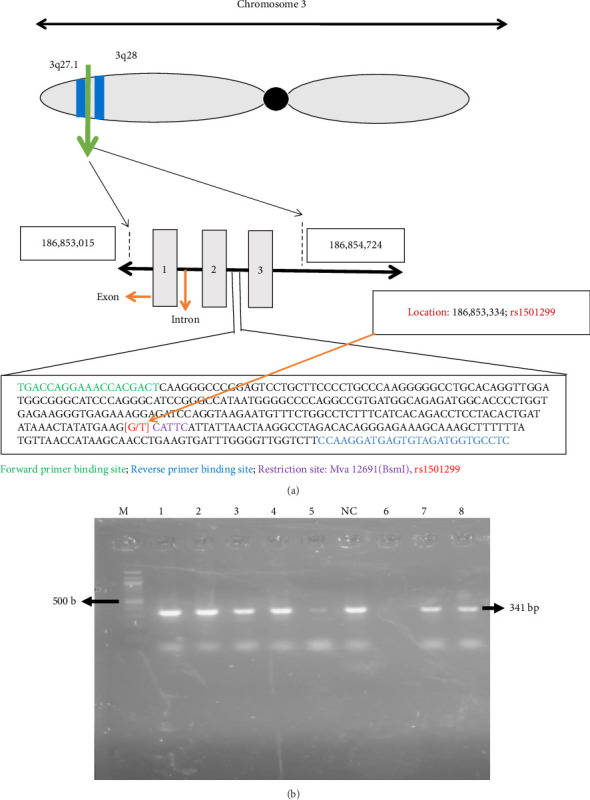
(a) Physical map of *ADIPOQ* rs1501299 with *Mva*12,691(*BsmI*) restriction site. The long arm of human chromosome 3 contains the *ADIPOQ* gene at a position between 3q27.1 and 3q28 (occupied on 186,853,314-186,853,353 location). The SNP rs1501299 is located in between 2^nd^ and 3^rd^ exons (location; 186,853,334). Exons are indicated as silver colored boxes. Introns are indicated in linear line. Nucleotides marked in green color indicate the binding site of forward primer, Nucleotides in blue color denote the binding site of reverse primer, indigo color nucleotides indicate restriction site of *Mva*12,691(*BsmI*) and red color nucleotide represents rs1501299 of *ADIPOQ* gene. (b) Amplification of *ADIPOQ* gene. PCR products were resolved on a 2.5% agarose gel with 100 bp ladder (Lane M) and PCR products containing *ADIPOQ* gene (341 bp) in Lanes 1–6, 7-8 and negative control (NC).

Adiponectin, a protein encoded by the *ADIPOQ* gene situated in the chromosomal region 3q27, plays a significant role in diabetes development. The *ADIPOQ* gene spans a length of 16 kilo-bases and is comprised of 2 introns and 3 exons encoding for the 30 kDa adiponectin protein [23], located on the long arm of human chromosome 3, at position between 3q27.1 and 3q28 (occupied on 186,853,015–186,854,724 location). The intron situated between the 2^nd^ exon and the 3^rd^ exon carries SNPrs1501299 (location; 186,853,334) (Figure 1(a)). A direct association was observed between the emergence of insulin resistance and T2DM and reduced serum adiponectin levels [24]. Numerous single nucleotide polymorphisms (SNPs) within the *ADIPOQ* gene have been documented as having a connection with the onset of T2DM [25, 26]. The rs1501299 is one of the polymorphisms in the *ADIPOQ* gene (SNP276) at position 276 is associated with insulin resistance and T2DM in the Saudi population [27]. Similar associations between the rs1501299 variant in the *ADIPOQ* gene and insulin resistance, as well as susceptibility to T2DM, have been documented in various ethnic groups [28–30]. The rs1501299 variant alone, or in conjunction with rs2241766 as a haplotype in exon 2, has been identified as being linked to obesity and insulin resistance [23, 26]. Additionally, individuals with impaired glucose metabolism carrying the T-allele of SNP rs1501299 face an elevated risk of progressing to T2DM [30]. For the rs1501299 polymorphism, G allele is reported to be as reference allele whereas T allele is reported to be alternative allele in all tested global population. Surprisingly, no C allele is reported till date for the rs1501299 in any of the tested population according to NCBI dbSNP database information [31]. Our current study also did not find any C allele for rs1501299 in both T2DM patient and control sample. According to NCBI dbSNP database, the global prevalence of G and T allele of rs1501299 was 0.730263 and 0.269737, respectively. Among the Asian population, the prevalence was found as G (0.7291) and T (0.2709), whereas the prevalence of G and T allele was 0.841 and 0.159 in South Asian population [31]. These findings echo those reported in other ethnic groups and emphasize the complex interplay of genetic factors in influencing metabolic outcomes, especially in relation to adiponectin and its implications for insulin resistance and T2DM risk.

To the best of our current understanding, no prior investigation has been conducted to assess the association between *ADIPOQ* rs1501299 variant and T2DM within the Bangladeshi population. Therefore, this study endeavors to bridge this research gap by scrutinizing the potential correlation of rs1501299 with the susceptibility to T2DM among individuals residing in the Noakhali region of Bangladesh. Furthermore, our investigation extends to exploring the connection between rs1501299 and various anthropometric and metabolic traits in individuals affected by T2DM. This study contributes novel insights into the genetic underpinnings of T2DM within the specific demographic context of the Noakhali region, thus advancing our understanding of the interplay between *ADIPOQ* rs1501299 and diabetes-related factors.

## 2. Methodology

### 2.1. Ethical Statement

The objectives of the study, the confidentiality measures, the participants' right to withdraw their involvement and their obligations were comprehensively elucidated to all study participants. Prior to the evaluation, each patient and healthy control voluntarily provided written informed consent. The research protocol for this study was officially sanctioned by the Ethical Clearance Committee within the Science Faculty of Noakhali Science and Technology University (Ethical Code: NSTU/SCI/EC/2022/102(A)). The entire genetic analysis was conducted at the Molecular Biology Laboratory, situated in the Department of Biotechnology and Genetic Engineering at Noakhali Science and Technology University in Bangladesh.

### 2.2. Study Design and Subject Recruitment

This case-control study involved 152 T2DM patients and 118 age- and gender-matched healthy volunteers. T2DM patients were selected from Al-Haj Sirajul Islam Diabetic and General Hospital, Maijdee, Noakhali, Bangladesh following the World Health Organization (WHO) criteria for diabetes diagnosis (fasting blood glucose or FBG level > 7.0 mmol/L or random plasma glucose level > 11.1 mmol/L). Detailed physical (including sex, age and body mass index or BMI) and clinical data such as FBG level, 2 h postprandial blood glucose level, blood pressure, total cholesterol (TC), triglycerides (TG), HDL and LDL were collected by trained nurses and expert physicians between February 2022 and September 2022. Healthy controls, meticulously matched for sex, age and BMI were selected from various locations in Noakhali. Their FBG levels were confirmed using a portable glucometer. Inclusion criteria for controls included normal glucose tolerance, no family history of severe diseases and the absence of other chronic illnesses.

In this study, all individuals with T2DM underwent comprehensive CVD analysis. Among these patients, it was observed that 82 individuals (53.95%) exhibited at least one of the following pathological conditions: diabetic cardiomyopathy, heart failure or coronary heart disease. These conditions collectively constituted T2DM-related CVD. All relevant clinical information was thoroughly documented through a questionnaire, and the analysis conducted adhered to the principles outlined in the Helsinki Declaration, including its subsequent amendments, to ensure ethical research practices [32].

### 2.3. Sampling and Biochemical Assay

As per standard procedures, a proficient nurse collected 3 mL of venous blood from both patients and healthy controls, and preserved these samples in EDTA-Na_2_ (Ethylenedinitrilotetra acetic acid disodium salt) containing sterile tubes. The collected blood samples were split into two microcentrifuge tubes. The first set was stored at −20°C for subsequent DNA extraction, aimed at SNP genotyping. The second set underwent centrifugation at 12,000 rpm for 10 min to isolate serum, which was then securely labeled and preserved at −20°C. This isolated serum was preserved for the analysis of TC and TG levels using spectrophotometric methods and commercially available kits from Human-GmbH, Germany. To ensure accuracy, all assays were performed in triplicate.

### 2.4. Molecular Genotyping

Genomic DNA was extracted from whole blood samples of both patients and a control group using the FavorPrep Blood Genomic DNA Extraction Mini Kit (Favorgen Biotech Corp, Taiwan). The extracted genomic DNA was subsequently employed for the specific amplification of a 341-bp region within the *ADIPOQ* gene through the polymerase chain reaction (PCR). The DNA extracts were stored at −20°C for preservation.

To genotype the *ADIPOQ* rs1501299 polymorphism, Restriction Fragment Length Polymorphism (RFLP) analysis was conducted. The PCR experiments were performed using the MiniAmp Plus Thermal Cycler (Applied Biosystems). Amplification was done by using PCR Master Mix (2X) from Promega Corporation, along with the following primers (Forward Primer: 5′-TGACCAGGAAACCACGACTC-3′ and Reverse Primer: 5′-CCATCTACACTCATCCTTGG-3′) as described for analyzing the association of rs1501299 in other populations [30, 33]. The PCR conditions involved an initial denaturation at 95°C for 5 min, followed by 35 cycles of (a) denaturation at 95°C for 1 min, (b) annealing at 53°C for 1 min and (c) elongation at 72°C for 1:30 min, concluding with a final elongation step at 72°C for 5 min.

Subsequently, the amplified DNA fragments underwent restriction digestion using *Mva*12691*(BsmI*) enzyme (New England Biolabs, England). The digestion was carried out at 37°C overnight. The reaction mixture (20.2 μL) for restriction digestion contained 15 μL of PCR product, 2 μL of 10X Tango buffer, 0.2 μL of *Mva*12691(*Bsm*I) enzyme and 3 μL of nuclease-free water. The digested PCR products were then separated by electrophoresis on a 2.5% agarose gel stained with ethidium bromide. The results were visualized under a UV transilluminator.

### 2.5. Statistical Analysis

In the study, various demographic data of patients with T2DM, such as age, BMI, FBG levels, 2 h postprandial blood glucose levels, blood pressure, TC, TG, HDL and LDL were compared to data from healthy controls. These comparisons were made using the one-way analysis of variance (ANOVA) test. Additionally, the study assessed the Hardy–Weinberg equilibrium (HWE) values through the HWE test (https://gene-calc.pl/hardy-weinberg-page).

To determine the genotypic associations of several SNPs with T2DM, various genetic models, including the heterozygote model (Aa vs. AA), homozygote model (aa vs. AA), dominant model (Aa + aa vs. AA), over dominant model (AA + aa vs. Aa), recessive model (AA + Aa vs. aa), and allele model (a vs. A), were examined. The study also presented the risk of T2DM and T2DM-related CVD in terms of adjusted odds ratios (OR). These OR values were adjusted based on factors such as age, gender, and BMI. The 95% confidence intervals (95% CI) were determined using the *χ*^2^-test and were evaluated using MedCalc software (https://www.medcalc.org/calc/odds_ratio.php). All calculations, including those related to HWE, were carried out using SPSS (Version 25, IBM). A significance level of *p* < 0.05 was employed for all analyses within this study.

## 3. Results

### 3.1. Characteristics of the Participants

The anthropometric, biochemical and demographic characteristics of the study populations are detailed in Table 1 and Supporting Tables 1 and 2. In the patient group, comprising 152 individuals diagnosed with T2DM, 54 (35.53%) were male, and 98 (64.47%) were female. On the other hand, the control group, consisting of 118 individuals without T2DM, showed that 71 (60.17%) were male, and 47 (39.83%) were female.

This study showed substantial disparities in terms of systolic blood pressure (SBP), diastolic blood pressure (DBP), TC, HDL, LDL and TG levels when comparing individuals with T2DM to the healthy control group (Table 1). These disparities in clinical features could be contributed by the age difference of the studied population along with T2DM and related complications. Another underlying cause may be the genetic association of the individuals. Additionally, the gender distribution, BMI, FBG, SBP, DBP were not significantly different between T2DM with CVD patients and T2DM without CVD. However, the levels of TC, HDL, LDL and TG were considerably different between T2DM with CVD patients and T2DM without CVD (Table 2).

### 3.2. The Impact of *ADIPOQ* SNP rs1501299 on T2DM

**Figure 2:**
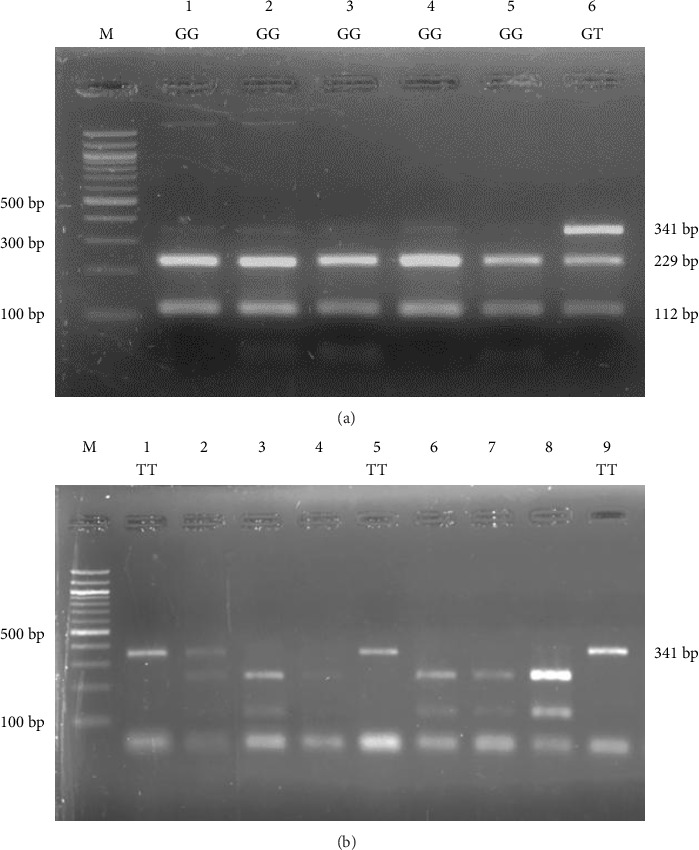
Genotyping of *ADIPOQ*-rs1501299 among T2DM patients using the *Mva*12691(*BsmI*) restriction enzyme. (a) Lane M displayed a 100 bp ladder marker. Lanes 1, 2, 3, 4 and 5 showed the (GG) form with distinct bands at 112 bp and 229 bp. Lane 6 revealed the (GT) form, displaying bands at 112 bp, 229 bp and 341 bp. (b) Lanes 1, 5 and 9 displayed the (TT) form with a single band at 341 bp. DNA fragments were separated by electrophoresis on a 2.5% agarose gel.

A successful PCR amplification of the specific *ADIPOQ* fragment containing the rs1501299 polymorphism was achieved for both the patient and control samples. When visualized, the PCR reaction product of a 341-base pair DNA band was produced (Figure 1(b)). Upon treating the PCR product with the *Mva*12691(*BsmI*) enzyme, diverse configurations of DNA bands were detected and are comprehensively outlined in Table 3 and Supporting Tables 1 and 2, along with a visual representation provided in Figure 2. The table provides a concise overview of the fragment sizes generated by the enzyme for each genetic type—wild, heterozygous and mutant—facilitating a clear understanding of the *ADIPOQ* gene variations.

In both the case and control groups, the conformity of genotype frequencies in all participants with the principles of HWE and ascertained marginal deviations from HWE for the genotypes was assessed and represented in Table 4.

In the patient group, the distribution of genotypes (GG-GT-TT) was 58.55%, 35.53%, and 5.92%, respectively. In the control group, the corresponding genotypes were 59.32%, 38.14%, and 2.54%. The *p* values for T2DM patients (*X*^2^ = 0.045, *p* = 0.978) and for control samples (*X*^2^ = 1.86138, *p* = 0.39428) are both higher than the commonly accepted threshold of 0.05 (*p* < 0.05), indicating consistence with HWE. This means that the observed frequencies of these genotypes are significantly different from what would be expected under the assumptions of HWE.

Furthermore, when assessing the impact of the *ADIPOQ* rs1501299 polymorphism on T2DM, we examined dominant, over dominant, recessive and allele models. After assessing the impact of the *ADIPOQ* rs1501299 polymorphism on T2DM patients, we did not find any significant association between the *ADIPOQ* rs1501299 polymorphism and T2DM at any genetic model (*p* value more than 0.05). On the other hand, according to the allelic model, we found that T allele showed a significant association for the impact of the *ADIPOQ* rs1501299 polymorphism on T2DM, with a *p* value less than 0.05 (OR: 1.8075, 95% CI: 1.1818 to 2.7644, *p*=0.0063). The OR and corresponding *p* values for each model are presented in Table 5.

### 3.3. Genotype Frequencies, HWE and Association With T2DM Risk

The genotype frequencies in our study population were determined to be GG (58.55%), GT (35.53%) and TT (5.92%). To assess conformity with HWE, expected frequencies were calculated based on the population allele frequencies. The expected frequencies under HWE were estimated using the Hardy–Weinberg equation, resulting in the following expected values: GG (58.24%), GT (36.15%), and TT (5.61%). The chi-squared test indicated no significant association in all genotypic models as suggested by the *p* value of HWE analysis (*p* < 0.05) (Table 6).

In case of dominant model, the OR for T2DM in the GT + TT genotype was 1.0323 (95% CI: 0.6331–1.6832), with a nonsignificant *p* value of 0.8986 (Table 5). This result suggests that the genotype combination GT + TT in dominant model may not conform to (HWE) in our study population. Similarly, the recessive model was analyzed where the OR for T2DM in the TT genotype was 2.4126 (95% CI: 0.6384–9.1178) with a nonsignificant *p* value of 0.1942 (Table 5). The chi-squared test revealed a significant deviation from HWE suggesting that the TT genotype may not adhere to HWE within our study population. GT genotype did not show any association with T2DM risk compared with GG + TT genotype with an OR ratio of 0.8939 (95% CI 0.5431–1.4713) in over dominant model and the result was not statistically significant (*p* = 0.6590) (Table 5).

Upon investigation of G and T allele frequencies, the G allele was observed at 76.32% in T2DM cases and 78.39% in healthy controls, while the T allele was present at 23.68% in cases and 21.61% in controls. A chi-squared test indicated a significant difference in T allele frequencies between cases and controls (*p* = 0.0063), suggesting potential influences on the T allele, such as selective or demographic factors. The OR for T2DM associated with the T allele was 1.8075 (95% CI: 1.1818–2.7644), with a significant *p* value of 0.0063, indicating a potential association between the T allele and T2DM susceptibility (Table 5).

### 3.4. Influences of rs1501299 With CVD in T2DM Patients

In both the T2DM-without-CVD groups and T2DM-with-CVD groups, the conformity of genotype frequencies in all cases with the principles of HWE and ascertained marginal deviations from HWE for the genotypes was assessed and represented in Table 7.

In the T2DM-with-CVD group, the distribution of genotypes (GG-GT-TT) was 66.102%, 28.814%, and 5.085%, respectively. In the T2DM-without-CVD group, the corresponding genotypes were 53.763%, 39.785%, and 6.452%. The *p* values for T2DM with CVD groups (*X*^2^ = 0.39596, *p* = 0.82038) and T2DM without CVD groups (*X*^2^ = 0.05894, *p* = 0.97096) are both higher than the commonly accepted threshold of 0.05 (*p* > 0.05), indicating consistence with HWE (Table 7). This means that the observed frequencies of these genotypes are significantly different from what would be expected under the assumptions of HWE.

To assess conformity with HWE, expected frequencies were calculated based on the population allele frequencies. The expected frequencies under HWE were estimated using the Hardy–Weinberg equation, resulting in the following expected values: GG (64.816%), GT (31.385%), and TT (3.799%) (Table 8). The Chi-squared test indicated a significant deviation from HWE (*p* < 0.05) which indicated that there is no significant association in any genotypic model within our study population.

In case of dominant model, the OR for T2DM-with-CVD group in the GT + TT genotype was 0.5963 (95% CI: 0.3034–1.1721), with a nonsignificant *p* value of 0.1338 (Table 9). This investigation also suggests that the genotype combination GT + TT in dominant model may not conform to HWE in our study population. Similarly, the recessive model was analyzed where the OR for T2DM-with-CVD group in the TT genotype was 0.7768 (95% CI: 0.1866–2.2331), with a nonsignificant *p* value of 0.7285 (Table 9). The chi-squared test revealed a significant deviation from HWE (*p* < 0.05), suggesting that the TT genotype may not adhere to HWE within our study population. GT genotype did not show any association with risk of T2DM-related CVD in T2DM patients compared with GG + TT genotype with an OR ratio of 0.6126 (95% CI 0.3042–1.2335) in over dominant model, with a nonsignificant *p* value of 0.1700 (Table 9). Upon investigation of G and T allele frequencies, the G allele was observed at 80.51% in T2DM with CVD cases and 73.66% in T2DM without CVD cases, while the T allele was present at 19.49% in T2DM with CVD cases and 26.34% in T2DM without CVD cases. A chi-squared test indicated a nonsignificant difference in T allele frequencies between these two groups (*p* = 0.1722), suggesting no significant association (OR: 0.6769; 95% CI: 0.3865–1.1854) of the T allele in T2DM-related CVD (Table 9). After the examination for the link of T2DM-related CVD in *ADIPOQ* rs1501299 SNP in T2DM patients, it was found that *ADIPOQ* rs1501299 did not show any substantial association in any evaluated genetic models in the studied population.

## 4. Discussion

T2DM is a significant medical issue and one of the most prominent noninfectious illnesses in the world. It is thought that T2DM is caused by a variety of intricate genetic variables [34–38]. Thus, it is crucial to look into these genetic variants that are linked to the risk of developing T2DM. The investigation conducted in this study sought to elucidate the potential association between the *ADIPOQ* rs1501299 polymorphism and the susceptibility to T2DM within the population of the Noakhali region in Bangladesh. With the rising global prevalence of T2DM, particularly in low to middle-income countries like Bangladesh, understanding the genetic underpinnings of this complex condition becomes crucial for devising effective preventive and therapeutic strategies.

This study revealed a significant association between the *ADIPOQ* rs1501299 polymorphism and T2DM. Notably, individuals carrying the T allele exhibited a higher risk of developing T2DM compared to those with the G allele. The association held true in both dominant and allele models, emphasizing the potential role of this genetic variant in influencing T2DM susceptibility within the studied population. These findings are consistent with previous research documenting associations between the rs1501299 variant in the adiponectin gene and insulin resistance, as well as the predisposition to T2DM, in various ethnic groups [29, 30]. The deviation from HWE for the TT genotype in our study population warrants consideration. This deviation may be indicative of underlying factors influencing the distribution of this genotype, such as selective pressures or demographic dynamics. Further, exploration into these factors could provide valuable insights into the genetic landscape of T2DM in the Noakhali region.

This investigation extended its focus to scrutinize both the clinical and biochemical characteristics of individuals diagnosed with T2DM in comparison to a control group consisting of healthy individuals. The disparities identified in several key parameters, including SBP, DBP, TC and TG levels, illuminate the intricate interplay between genetic factors, metabolic outcomes, and CVD among those affected by T2DM. Notably, our findings reveal distinct differences in SBP and DBP, highlighting the impact of T2DM on blood pressure regulation. Additionally, variations in TC and TG levels suggest a profound influence of the disease on lipid metabolism. These observed disparities underscore the complex relationship between genetic factors and the physiological manifestations of T2DM, emphasizing the importance of understanding the multifaceted nature of the condition [39, 40].

Furthermore, the heightened prevalence of CVD among individuals within our T2DM patient cohort serves as a critical point of consideration. This underscores the imperative for comprehensive risk assessment and the development of tailored management strategies that account for both the genetic predispositions and clinical characteristics unique to individuals in this population. The need for a nuanced and individualized approach becomes particularly apparent in addressing the heightened cardiovascular risk associated with T2DM, necessitating targeted interventions that consider the specific genetic and clinical profiles of affected individuals. A favorable correlation was found between an elevated risk of coronary artery disease (CAD) and the *ADIPOQ* (rs1501299) mutation in a study on the East Asian population [41]. According to a research on the Iranian study population, the T allele of SNP rs1501299 and the GT and TT genotypes were linked to an elevated risk of CAD [42]. Additionally, it was noticed that the frequency of the T allele was substantially higher in female CAD patients than in control subjects [42]. Amrita et al. reported that the frequency of rs1501299 T allele carriers was significantly higher in women with CVD as compared to women without CVD [29]. However, heterozygosity at *rs*1501299 polymorphism was shown to provide protection against CVD, whereas, this SNP was found not to be associated with CVD in homozygote GG model [29]. Conversely, rs1501299 was substantially marked as a low-risk factor for the development of CVD with T2DM [43], although no association of this same *ADIPOQ* SNP was also reported [44]. Moreover, *ADIPOQ* rs1501299 polymorphism was suggested to play a protective role in CAD in diabetic patients in homozygote, allelic and recessive models [45]. However, in our study population, no substantial association was found between *ADIPOQ* rs1501299 of T2DM with and without CVD in any evaluated genetic models. The link between the *ADIPOQ* rs1501299 polymorphism and the susceptibility to T2DM has been confirmed in specific population groups. Specifically, individuals with the GG genotype exhibit lower plasma adiponectin levels, increased insulin resistance, and a heightened susceptibility to developing T2DM compared to those with the TT genotype in various population [26, 27]. This intriguing phenotype has been observed consistently in various ethnic groups, underscoring the significance of rs1501299 in influencing plasma adiponectin levels, insulin resistance, and T2DM risk [23, 27, 28]. Furthermore, investigations in various other population revealed a linkage between rs1501299, either alone or in conjunction with rs2241766, and obesity as well as insulin resistance and diabetes [46–48]. Individuals with impaired glucose tolerance and the T-allele of SNP rs1501299 are at an elevated risk of progressing to T2DM. Despite the well-established benefits of regular exercise for individuals with T2DM, there is no discernible correlation between the impact of twice-weekly exercise training and the adiponectin gene polymorphism rs1501299 on total and high molecular weight adiponectin levels [49].

While providing valuable insights, it is essential to acknowledge certain limitations that warrant careful consideration. First and foremost, the sample size in our study may be a limiting factor, potentially affecting the generalizability of our findings. The restricted number of participants might not fully capture the genetic diversity inherent in the broader population and caution is advised when extrapolating our results to other regions or ethnic groups. Furthermore, the regional focus on the Noakhali population may introduce a degree of specificity that could limit the broader applicability of our conclusions. Genetic and environmental factors can vary significantly across different geographical areas and caution should be exercised when extending our observations to populations with distinct demographic characteristics. To enhance the robustness and generalizability of our observations, future studies should aim for larger and more diverse cohorts that encompass various demographic and ethnic backgrounds. Furthermore, delving into the functional implications of the *ADIPOQ* rs1501299 polymorphism presents an avenue for deeper understanding. Investigating its impact on adiponectin levels and downstream effects could furnish mechanistic insights into its specific role in the pathogenesis of T2DM. Such exploration would not only validate our current findings but also contribute to a more comprehensive understanding of the molecular mechanisms at play.

In the broader context, our study adds a significant layer to the expanding body of evidence linking genetic factors, particularly the *ADIPOQ* rs1501299 polymorphism, to T2DM susceptibility. The emphasis on genetic variations underscores the necessity of integrating such factors into the realm of personalized medicine for both risk assessment and management of T2DM. This recognition prompts a shift towards more tailored approaches that consider an individual's genetic makeup in the formulation of preventive and therapeutic strategies. Looking ahead, further research endeavors should aim to unravel the functional significance of these genetic associations. Understanding how these variations influence the development and progression of T2DM at a molecular level could open avenues for targeted and more effective therapeutic interventions. This holistic approach holds promise in refining our strategies for tackling the global T2DM epidemic, moving us closer to precision medicine solutions.

## 5. Conclusion

T2DM is largely known to be influenced by genetic factors. The current study reveals a significant association between the *ADIPOQ* rs1501299 polymorphism and T2DM in the Noakhali region of Bangladesh. The heightened risk of T2DM conferred by the rs1501299 T allele emphasizes the importance of genetic factors in disease susceptibility. The deviation from HWE for the TT genotype suggests unique demographic influences on T2DM genetics in this population. Clinical disparities identified for various parameters in T2DM patients underscore the intricate interplay between genetics and metabolic outcomes. *ADIPOQ* rs1501299 of T2DM with/without CVD did not show any significant association in any evaluated genetic models in our study population. Despite having a few limitations, this study serves as a valuable starting point for understanding the genetic underpinnings of T2DM in the population of Noakhali region. Our study indicates the significance of precision medicine in T2DM, underscoring the necessity for personalized risk assessments. By unraveling the complexities of T2DM genetics, our findings lay a robust foundation for tailored interventions. This emphasizes the shift away from generic approaches, fostering a more nuanced and effective strategy to address the diverse clinical and genetic profiles within the T2DM patient population. The implications of our research extend to optimizing therapeutic outcomes and contributing to more precise and individualized management of this global health challenge.

## Figures and Tables

**Table 1:** Anthropometric, biochemical and demographic features of the study subjects.

Characteristics	T2DM (*n* = 152)	Control (*n* = 118)
Gender (M (*n*%)/F (*n*%))	54 (34.53)/98 (64.47)	71 (60.17)/47 (39.83)
Age (years) of onset	48.55 (±11.21)	—
Age at examination (years)	54.56 (±10.94)	37 (±6.02)
Body mass index (kg/m^2^)	25.55 (±7.07)	26.1 (±5.80)
Smoking (*n* %)	13 (6.05)	2 (3.27)
Systolic blood pressure (mmHg)	134.98 (±17.3)	115 (±7.07)
Diastolic blood pressure (mmHg)	79.97 (±11.06)	70 (±7.07)
CVD (*n* %)	59 (34.21)	0
FBG (mmol/L)	9.83 (±3.79)	< 6.9
2HPBG (mmol/L)	14.54 (±4.97)	< 7.9
RBG (mmol/L)	12.16 (±4.79)	< 7
Total cholesterol (mg/dL)	231.74 (±7.48)	176.59 (±6.49)
Triglycerides (mg/dL)	193.03 (±14.17)	133 (±15.06)
HDL cholesterol (mg/dL)	24.42 (±4.55)	48 (±11.32)
LDL cholesterol (mg/dL)	170.23 (±10.33)	102 (±15.87)

*Note:* Data are expressed as mean ± SD.

Abbreviations: 2HPBG, 2 h postprandial blood glucose; CVD, cardiovascular disease; FBG, fasting blood glucose; HDL, high-density lipoprotein; LDL, low-density lipoprotein; RBG: random blood glucose.

**Table 2:** Anthropometric, biochemical and demographic features of T2DM with CVD and T2DM without CVD patients.

Demographic characteristics	T2DM with CVD (*n* = 59) (%)	T2DM without CVD (*n* = 93) (%)
Gender (male/female) (*n* %)	23 (38.99)/36 (61.02)	31 (33.33)/62 (66.67)
Age (years) of onset	47.86 (±12.68)	48.98 (±10.28)
Age at examination (years)	53.86 (±10.9)	55 (±10.98)
Body mass index (kg/m^2^)	24.10 (±5.7)	21.3 (±5.3)
Smoking (*n* %)	5 (8.5)	7 (7.5)
Medication of blood pressure (*n* %)	59 (100)	93 (100)
Systolic blood pressure (mmHg)	133.62 (±7.03)	115 (±17.87)
Diastolic blood pressure (mmHg)	75.97 (±7.06)	75 (±11.28)
FBG (mmol/L)	9.51 (±0.87)	8.34 (±3.76)
2HPBG (mmol/L)	16.31 (±3.31)	16.43 (±1.72)
RBG (mmol/L)	10.29 (±3.23)	9.55 (±0.87)
Total cholesterol (mg/dL)	245.4 (±7.84)	212.6 (±8.49)
Triglycerides (mg/dL)	202.5 (±12.5)	168.5 (±15.45)
HDL cholesterol (mg/dL)	21.75 (±7.72)	32.5 (±10.61)
LDL cholesterol (mg/dL)	184.62 (±14.05)	151 (±16.87)

*Note:* Data are expressed as mean ± SD.

Abbreviations: 2HPBG, 2 h postprandial blood glucose; BMI, body mass index; CVD, cardio vascular disease; FBG, fasting blood glucose; HDL, high-density lipoprotein; LDL, low-density lipoprotein; RBG: random blood glucose.

**Table 3:** Restriction digestion of PCR products of *ADIPOQ* gene with *Mva*12691(*BsmI*) enzyme.

Genotype	Fragment size (bp)
GG	229, 112
GT	341, 229, 112
TT	341

**Table 4:** Genotype distribution and HWE test of *ADIPOQ*-rs1501299 among T2DM patients and controls.

*ADIPOQ* rs1501299	T2DM (%) (*n* = 152)		*X* ^2^	*p* value	Controls (%) (*n* = 118)		*X* ^2^	*p* value
	M (%)	F (%)			M (%)	F (%)		
GG	89 (58.55)		0.045	0.978	70 (59.32)		1.86138	0.39428
	37 (24.34)	52 (34.21)			40 (33.90)	30 (25.42)		
GT	54 (35.53)				45 (38.14)			
	17 (11.18)	37 (24.34)			28 (23.73)	17 (14.41)		
TT	9 (5.92)				3 (2.54)			
	0	9 (5.92)			3 (2.54)	0		

*Note:* If *p* > 0.05, it is consistent with Hardy–Weinberg equilibrium.

**Table 5:** *ADIPOQ* rs1501299 genotypes among T2DM patients and control individuals and their association with diabetes.

*ADIPOQ* rs1501299	T2DM (%) (*n* = 152)	Controls (%) (*n* = 118)	OR (95% CI)	*p* value
GG	89 (58.55)	70 (59.32)	Reference	—
GT	54 (35.53)	45 (38.14)	0.8939 (0.5431–1.4713)	0.6590
TT	9 (5.92)	3 (2.54)	2.4126 (0.6384–9.1178)	0.1942

*Dominant model (GT + TT vs. GG)*				
GG	89 (58.55)	70 (59.32)	Reference	—
GT + TT	63 (41.45)	48 (40.68)	1.0323 (0.6331–1.6832)	0.8986

*Recessive model (TT vs. GG + GT)*				
GG + GT	143 (94.08)	115 (97.46)	Reference	—
TT	9 (5.92)	3 (2.54)	2.4126 (0.6384–9.1178)	0.1942

*Over dominant model (TT + GG vs. GT)*				
TT + GG	98 (64.47)	73 (61.86)	Reference	—
GT	54 (35.53)	45 (38.14)	0.8939 (0.5431–1.4713)	0.6590

*Allele model*				
G allele	232 (76.32)	185 (78.39)	Reference	—
T allele	72 (23.68)	51 (21.61)	1.8075 (1.1818–2.7644)	^∗^0.0063

Abbreviations: 95% CI, 95% confidence intervals; OR, odds ratio.

^∗^Here, if *p* < 0.05, it is considered as statistically significant result.

**Table 6:** Chi-squared test for deviation from HWE in genotype frequencies.

Genotype	Observed frequency (O)	Expected frequency (E) (under HWE)	*p* value (O−E)^2^/E
GG	58.55%	58.24%	< 0.05
GT	35.53%	36.15%	
TT	5.92%	5.61%	

**Table 7:** Genotypic distributions of *ADIPOQ* rs1501299 for T2DM-with-CVD group and T2DM without-CVD group.

Genotypes	T2DM with CVD (*n* = 59) %	Hardy–Weinberg equilibrium (HWE)		T2DM without CVD (*n* = 93) %	Hardy–Weinberg equilibrium (HWE)	
		*X* ^2^	*p* value		*X* ^2^	*p* value
GG	39 (66.102)	0.39596	0.82038	50 (53.763)	0.05894	0.97096
GT	17 (28.814)			37 (39.785)		
TT	3 (5.085)			6 (6.452)		

*Note:* If *p* > 0.05, it is consistent with Hardy–Weinberg equilibrium.

**Table 8:** Chi-squared test for deviation from HWE in genotype frequencies.

Genotype	Observed frequency (O)	Expected frequency (E) (under HWE)	*p* value (O−E)^2^/E
GG	66.102%	64.816%	< 0.05
GT	28.814%	31.385%	
TT	5.085%	3.799%	

**Table 9:** Association of alleles or genotypes with risk of T2DM-related CVD in T2DM patients.

Genotypes	T2DM with CVD (*n* = 59) %	T2DM without CVD (*n* = 93) %	OR (95% CI)	*p* value
GG	39 (66.102)	50 (53.763)	Reference	*—*
GT	17 (28.814)	37 (39.785)	0.6126 (0.3042–1.2335)	*p* = 0.1700
TT	3 (5.085)	6 (6.452)	0.7768 (0.1866–3.2331)	*p* = 0.7285

*Dominant model*				
GG	39 (66.102)	50 (53.763)	Reference	*—*
GT + TT	20 (33.898)	43 (46.237)	0.5963 (0.3034–1.1721	*p* = 0.1338

*Recessive model*				
GG + GT	56 (94.915)	87 (93.548)	Reference	*—*
TT	3 (5.085)	6 (6.452)	0.7768 (0.1866–3.2331)	*p* = 0.7285

*Over dominant model (TT + GG vs. GT)*				
TT + GG	42 (71.186)	56 (60.215)	Reference	*—*
GT	17 (28.814)	37 (39.785)	0.6126 (0.3042–1.2335)	*p* = 0.1700

*Allele model*				
G allele	95 (80.51)	137 (73.66)	Reference	*—*
T allele	23 (19.49)	49 (26.34)	0.6769 (0.3865–1.1854)	*p* = 0.1722

*Note:* Here, if *p* < 0.05, it is considered as statistically significant result

Abbreviations: 95% CI, 95% confidence intervals; OR, odds ratio.

## Data Availability

The data that supports the findings of this study are available in the Supporting Information of this article.

## Supporting Information

Supporting Table 1: Anthropometric, biochemical and genotypic (ADIPOQ (rs1501299) data of 118 healthy control samples (nondiabetic) from Noakhali region. A total of 118 individuals blood samples (who were nondiabetic and had no heart related disease) were collected from Noakhali region, Bangladesh. The male female ratio of the control samples was 71:47, and the average age was 37 (±6.02) years. The single nucleotide polymorphism (SNP) rs1501299 of ADIPOQ gene was examined through restriction fragment length polymorphism (RFLP) technique. Healthy individuals (control) and their blood samples were analyzed for their anthropometric, biochemical and genotypic (ADIPOQ rs1501299) examination.

Supporting Table 2: Anthropometric, biochemical and genotypic (ADIPOQ (rs1501299) data of 152 diabetic patients in Noakhali region. The blood samples were collected from medically diagnosed 152 diabetic patients and the patient's disease history (heart disease) was checked. The average age of the patients was 54.56 (±10.94) years, and the average age of onset of diabetes was 48.55 (±11.21) years. The male female ratio was 54:98. The SNP of (rs1501299) in ADIPOQ was investigated through RFLP method. Diabetic patients and their blood samples were analyzed for their anthropometric, biochemical and genotypic (ADIPOQ rs1501299) assessment.
