# Supplementary material for: Genetic Insights Into Type 2 Diabetes Mellitus Susceptibility: A Case-Control Study of the ADIPOQ rs1501299 Polymorphism in the Population of Noakhali Region of Bangladesh
Source: Genet Res (Camb). 2025 May 24;2025:8818420. doi: 10.1155/genr/8818420 (PMC12126263; doi:10.1155/genr/8818420)
Supplement: Supporting Information — Additional supporting information can be found online in the Supporting Information section. [file 8818420.f1.docx]

**Supplementary data (Raw data)**

**Supplementary Table 1:** Anthropometric, biochemical and genotypic (ADIPOQ rs1501299) data of 118 healthy control samples (non-diabetic) from Noakhali region.

| **Serial No.** | **Gender** | **Age** | **BMI** | **BP (mm/Hg)** | **Age of onset diabetes** | **FBS(mmol/L)** | **2HABF**  **(mmol\|L)** | **RBS**  **(mmol/L)** | **Presence of heart disease** | **Addiction to smoking** | **ADIPOQ (rs1501299)** |
| --- | --- | --- | --- | --- | --- | --- | --- | --- | --- | --- | --- |
| C-1 | Female | 21 | 30.2 | 110/70 | N.A. | N.A. | N.A. | N.A. | No | No | GG |
| C-2 | Male | 22 | 28.6 | 120/80 | N.A. | N.A. | N.A. | N.A. | No | No | GG |
| C-3 | Male | 40 |  | 120/80 | N.A. | N.A. | N.A. | N.A. | No | Yes | GG |
| C-4 | Male | 39 |  | 110/75 | N.A. | N.A. | N.A. | N.A. | No | No | GG |
| C-5 | Male | 41 |  | 120/80 | N.A. | N.A. | N.A. | N.A. | No | No | GG |
| C-6 | Male | 21 | 21.2 | 120/80 | N.A. | N.A. | N.A. | N.A. | No | No | GG |
| C-7 | Male | 45 |  | 120/80 | N.A. | N.A. | N.A. | N.A. | No | No | GT |
| C-8 | Male | 20 | 17.3 | 120/80 | N.A. | N.A. | N.A. | N.A. | No | No | TT |
| C-9 | Female | 21 | 23 | 110/70 | N.A. | N.A. | N.A. | N.A. | No | No | GT |
| C-10 | Female | 21 | 21.1 | 110/70 | N.A. | N.A. | N.A. | N.A. | No | No | GT |
| C-11 | Female | 31 | 23.4 | 120/80 | N.A. | N.A. | N.A. | N.A. | No | No | GT |
| C-12 | Male | 21 | 28.2 | 120/80 | N.A. | N.A. | N.A. | N.A. | No | No | GG |
| C-13 | Female | 29 | 24.2 | 120/80 | N.A. | N.A. | N.A. | N.A. | No | No | GG |
| C-14 | Male | 23 | 22.1 | 120/80 | N.A. | N.A. | N.A. | N.A. | No | No | GG |
| C-15 | Male | 50 |  | 110/80 | N.A. | N.A. | N.A. | N.A. | No | No | GT |
| C-16 | male | 24 | 23.1 | 120/80 | N.A. | N.A. | N.A. | N.A. | No | Yes | GT |
| C-17 | female | 22 | 22.7 | 120/80 | N.A. | N.A. | N.A. | N.A. | No | No | GG |
| C-18 | female | 23 | 17 | 120/80 | N.A. | N.A. | N.A. | N.A. | No | No | GG |
| C-19 | female | 22 | 15.2 | 120/80 | N.A. | N.A. | N.A. | N.A. | No | No | GG |
| C-20 | female | 22 | 23.8 | 120/80 | N.A. | N.A. | N.A. | N.A. | No | No | GT |
| C-21 | Male | 39 |  | 120/75 | N.A. | N.A. | N.A. | N.A. | No | No | GT |
| C-22 | female | 22 | 17.6 | 110/70 | N.A. | N.A. | N.A. | N.A. | No | No | GG |
| C-23 | female | 22 | 20.7 | 120/80 | N.A. | N.A. | N.A. | N.A. | No | No | GG |
| C-24 | male | 22 | 23.5 | 120/80 | N.A. | N.A. | N.A. | N.A. | No | No | GT |
| C-25 | male | 22 | 24.9 | 120/80 | N.A. | N.A. | N.A. | N.A. | No | No | GT |
| C-26 | male | 22 | 18.2 | 120/80 | N.A. | N.A. | N.A. | N.A. | No | No | GT |
| C-27 | male | 22 | 19.7 | 120/80 | N.A. | N.A. | N.A. | N.A. | No | No | GG |
| C-28 | male | 23 | 21.2 | 120/80 | N.A. | N.A. | N.A. | N.A. | No | No | GG |
| C-29 | male | 24 | 23.8 | 120/80 | N.A. | N.A. | N.A. | N.A. | No | No | GG |
| C-30 | male | 23 | 22.8 | 120/80 | N.A. | N.A. | N.A. | N.A. | No | No | GT |
| C-31 | Male | 21 | 19.2 | 120/80 | N.A. | N.A. | N.A. | N.A. | No | No | GG |
| C-32 | Male | 22 | 18 | 120/80 | N.A. | N.A. | N.A. | N.A. | No | No | GG |
| C-33 | Male | 23 | 24.6 | 120/80 | N.A. | N.A. | N.A. | N.A. | No | No | GG |
| C-34 | Male | 24 | 24.6 | 120/80 | N.A. | N.A. | N.A. | N.A. | No | No | GG |
| C-35 | Male | 26 | 24.5 | 120/80 | N.A. | N.A. | N.A. | N.A. | No | No | GT |
| C-36 | Male | 26 | 20.2 | 110/80 | N.A. | N.A. | N.A. | N.A. | No | No | GT |
| C-37 | Female | 26 | 24.1 | 115/70 | N.A. | N.A. | N.A. | N.A. | No | No | GG |
| C-38 | Male | 36 |  | 120/80 | N.A. | N.A. | N.A. | N.A. | No | No | GG |
| C-39 | Female | 26 | 20.1 | 110/70 | N.A. | N.A. | N.A. | N.A. | No | No | GT |
| C-40 | Female | 26 | 19.8 | 110/70 | N.A. | N.A. | N.A. | N.A. | No | No | GG |
| C-41 | Female | 26 | 18.7 | 115/70 | N.A. | N.A. | N.A. | N.A. | No | No | GG |
| C-42 | Male | 39 |  | 110/75 | N.A. | N.A. | N.A. | N.A. | No | No | GT |
| C-43 | Male | 47 |  | 120/80 | N.A. | N.A. | N.A. | N.A. | No | Yes | GT |
| C-44 | Male | 38 |  | 110/80 | N.A. | N.A. | N.A. | N.A. | No | No | GG |
| C-45 | Female | 25 | 28.4 | 115/65 | N.A. | N.A. | N.A. | N.A. | No | No | GG |
| C-46 | Male | 39 |  | 110/75 | N.A. | N.A. | N.A. | N.A. | No | No | GG |
| C-47 | Male | 24 | 22.4 | 120/80 | N.A. | N.A. | N.A. | N.A. | No | No | GT |
| C-48 | Male | 22 | 21.5 | 120/80 | N.A. | N.A. | N.A. | N.A. | No | No | GG |
| C-49 | Male | 25 | 25.3 | 120/80 | N.A. | N.A. | N.A. | N.A. | No | No | GG |
| C-50 | Male | 26 | 21.3 | 120/80 | N.A. | N.A. | N.A. | N.A. | No | No | GT |
| C-51 | Male | 26 | 27.6 | 120/80 | N.A. | N.A. | N.A. | N.A. | No | No | GG |
| C-52 | Female | 26 | 22.3 | 120/80 | N.A. | N.A. | N.A. | N.A. | No | No | GG |
| C-53 | Male | 38 | 22 | 120/80 | N.A. | N.A. | N.A. | N.A. | No | No | GT |
| C-54 | Male | 45 | 24 | 120/80 | N.A. | N.A. | N.A. | N.A. | No | No | GG |
| C-55 | Male | 43 | 21.9 | 130/90 | N.A. | N.A. | N.A. | N.A. | No | No | GG |
| C-56 | Male | 32 | 26.5 | 130/90 | N.A. | N.A. | N.A. | N.A. | No | No | GT |
| C-57 | Male | 52 | 22.7 | 120/80 | N.A. | N.A. | N.A. | N.A. | No | No | GG |
| C-58 | Female | 45 | 22 | 120/90 | N.A. | N.A. | N.A. | N.A. | No | No | GT |
| C-59 | Male | 38 | 24.5 | 120/80 | N.A. | N.A. | N.A. | N.A. | No | No | GG |
| C-60 | Male | 40 | 21.2 | 120/80 | N.A. | N.A. | N.A. | N.A. | No | No | GT |
| C-61 | Male | 40 | 22 | 120/80 | N.A. | N.A. | N.A. | N.A. | No | No | GG |
| C-62 | Female | 30 | 21.3 | 110/75 | N.A. | N.A. | N.A. | N.A. | No | No | GG |
| C-63 | Female | 37 | 22.3 | 120/80 | N.A. | N.A. | N.A. | N.A. | No | No | GG |
| C-64 | Male | 40 | 21 | 120/80 | N.A. | N.A. | N.A. | N.A. | No | No | GG |
| C-65 | Female | 50 | 24 | 120/80 | N.A. | N.A. | N.A. | N.A. | No | No | GG |
| C-66 | Male | 36 | 22 | 120/80 | N.A. | N.A. | N.A. | N.A. | No | No | GT |
| C-67 | Male | 29 | 22.3 | 120/80 | N.A. | N.A. | N.A. | N.A. | No | No | GG |
| C-68 | Male | 27 | 22.4 | 120/80 | N.A. | N.A. | N.A. | N.A. | No | No | GG |
| C-69 | Female | 36 | 21.5 | 120/80 | N.A. | N.A. | N.A. | N.A. | No | No | GT |
| C-70 | Female | 57 | 23.5 | 120/80 | N.A. | N.A. | N.A. | N.A. | No | No | GG |
| C-71 | Female | 25 | 22 | 130/90 | N.A. | N.A. | N.A. | N.A. | No | No | GT |
| C-72 | Female | 31 | 24.5 | 130/90 | N.A. | N.A. | N.A. | N.A. | No | No | GG |
| C-73 | Male | 43 | 23.4 | 120/80 | N.A. | N.A. | N.A. | N.A. | No | No | GG |
| C-74 | Female | 29 | 21.9 | 120/90 | N.A. | N.A. | N.A. | N.A. | No | No | GT |
| C-75 | Male | 42 | 24.3 | 120/80 | N.A. | N.A. | N.A. | N.A. | No | No | GG |
| C-76 | Male | 38 | 23.6 | 120/80 | N.A. | N.A. | N.A. | N.A. | No | Yes | GT |
| C-77 | Female | 20 | 19.4 | 125/85 | N.A. | N.A. | N.A. | N.A. | No | No | GG |
| C-78 | Male | 28 | 22.5 | 130/90 | N.A. | N.A. | N.A. | N.A. | No | No | GT |
| C-79 | Female | 32 | 21.2 | 130/90 | N.A. | N.A. | N.A. | N.A. | No | No | GG |
| C-80 | Male | 22 | 22 | 120/80 | N.A. | N.A. | N.A. | N.A. | No | No | GG |
| C-81 | Female | 20 | 21.3 | 120/90 | N.A. | N.A. | N.A. | N.A. | No | No | GT |
| C-82 | Female | 60 | 22.3 | 120/80 | N.A. | N.A. | N.A. | N.A. | No | No | GG |
| C-83 | Female | 54 | 21 | 120/80 | N.A. | N.A. | N.A. | N.A. | No | No | GG |
| C-84 | Male | 49 | 24 | 130/90 | N.A. | N.A. | N.A. | N.A. | No | No | GG |
| C-85 | Male | 32 | 22 | 130/90 | N.A. | N.A. | N.A. | N.A. | No | Yes | GT |
| C-86 | Female | 37 | 22.3 | 120/80 | N.A. | N.A. | N.A. | N.A. | No | No | GG |
| C-87 | Male | 20 | 22.4 | 120/80 | N.A. | N.A. | N.A. | N.A. | No | No | GG |
| C-88 | Male | 21 | 21.2 | 110/75 | N.A. | N.A. | N.A. | N.A. | No | No | GT |
| C-89 | Male | 26 | 22 | 120/80 | N.A. | N.A. | N.A. | N.A. | No | No | GG |
| C-90 | Female | 31 | 21 | 120/80 | N.A. | N.A. | N.A. | N.A. | No | No | GG |
| C-91 | Female | 48 | 24 | 120/80 | N.A. | N.A. | N.A. | N.A. | No | No | GG |
| C-92 | Female | 52 | 22 | 120/80 | N.A. | N.A. | N.A. | N.A. | No | No | GT |
| C-93 | Female | 37 | 22.3 | 120/80 | N.A. | N.A. | N.A. | N.A. | No | No | GT |
| C-94 | Female | 19 | 22.4 | 120/80 | N.A. | N.A. | N.A. | N.A. | No | No | GG |
| C-95 | Male | 29 | 21.2 | 125/85 | N.A. | N.A. | N.A. | N.A. | No | No | TT |
| C-96 | Male | 35 | 22 | 130/90 | N.A. | N.A. | N.A. | N.A. | No | No | GT |
| C-97 | Male | 34 | 21 | 130/90 | N.A. | N.A. | N.A. | N.A. | No | No | GT |
| C-98 | Male | 36 | 24 | 120/80 | N.A. | N.A. | N.A. | N.A. | No | No | GG |
| C-99 | Male | 33 | 22 | 120/90 | N.A. | N.A. | N.A. | N.A. | No | No | GG |
| C-100 | Male | 27 | 22.3 | 120/80 | N.A. | N.A. | N.A. | N.A. | No | No | GT |
| C-101 | Female | 36 | 22.4 | 120/80 | N.A. | N.A. | N.A. | N.A. | No | No | GG |
| C-102 | Female | 51 | 21.2 | 130/90 | N.A. | N.A. | N.A. | N.A. | No | No | GT |
| C-103 | Male | 38 | 22 | 130/90 | N.A. | N.A. | N.A. | N.A. | No | Yes | GT |
| C-104 | Female | 29 | 21 | 125/85 | N.A. | N.A. | N.A. | N.A. | No | No | GG |
| C-105 | Male | 41 | 24 | 130/90 | N.A. | N.A. | N.A. | N.A. | No | No | GT |
| C-106 | Male | 31 | 22.3 | 120/80 | N.A. | N.A. | N.A. | N.A. | No | Yes | GT |
| C-107 | Male | 37 | 24.6 | 120/90 | N.A. | N.A. | N.A. | N.A. | No | No | GG |
| C-108 | Female | 28 | 22.5 | 120/80 | N.A. | N.A. | N.A. | N.A. | No | No | GT |
| C-109 | Male | 45 | 21.5 | 120/80 | N.A. | N.A. | N.A. | N.A. | No | No | TT |
| C-110 | Male | 54 | 25.6 | 130/90 | N.A. | N.A. | N.A. | N.A. | No | No | GT |
| C-111 | Female | 70 | 22.5 | 130/90 | N.A. | N.A. | N.A. | N.A. | No | No | GG |
| C-112 | Male | 30 | 21 | 120/80 | N.A. | N.A. | N.A. | N.A. | No | No | GT |
| C-113 | Female | 43 | 24 | 120/80 | N.A. | N.A. | N.A. | N.A. | No | No | GT |
| C-114 | Male | 25 | 22 | 110/75 | N.A. | N.A. | N.A. | N.A. | No | No | GG |
| C-115 | Male | 50 | 22.3 | 130/90 | N.A. | N.A. | N.A. | N.A. | No | No | GG |
| C-116 | Female | 36 | 22.4 | 120/80 | N.A. | N.A. | N.A. | N.A. | No | No | GG |
| C-117 | Male | 49 | 21.2 | 120/90 | N.A. | N.A. | N.A. | N.A. | No | Yes | GG |
| C-118 | Female | 73 | 22 | 120/80 | N.A. | N.A. | N.A. | N.A. | No | No | GG |
|  |  |  |  |  |  |  |  |  |  |  |  |
|  |  |  |  |  |  |  |  |  |  |  |  |

**Supplementary Table 2:** Anthropometric, biochemical and genotypic (ADIPOQ rs1501299) data of 152 diabetic patients in Noakhali region.

| **Serial No.** | **Gender** | **Age** | **BMI(kg/m2)** | **BP(mm/Hg),**  **Using Drug for BP** | **Age at Onset of diabetes** | **FBS(mmol/L)** | **2HABF**  **(mmol\|L)** | **RBS**  **(mmol/L)** | **Presence of heart disease** | **Addiction to smoking** | **ADIPOQ (rs1501299)** |
| --- | --- | --- | --- | --- | --- | --- | --- | --- | --- | --- | --- |
| S-02 | Male | 45 | 17.54 | 110/70,yes | 45 | 8.34 | 17.64 |  | No | No | GG |
| S-03 | Male | 48 | 21.26 | 110/70,yes | 25 | 8.89 | 13.96 |  | Yes | No | GG |
| S-04 | Female | 46 | 22.72 | 110/65,yes | 43 | 8.58 | 16.94 |  | No | No | GT |
| S-05 | Female | 32 | 26.22 | 110/70,yes | 27 | 10.04 | 12.4 |  | No | No | GG |
| S-06 | Male | 55 | 20.9 | 110/70,yes | 40 |  |  | 13.28 | No | Yes | GG |
| S-08 | Male | 81 | 18.38 | 120/80,yes | 80 | 11.1 | 13.4 |  | Yes | No | GG |
| S-10 | Male | 51 | 21.34 | 120/80,yes | 36 |  |  | 10.85 | Yes | Yes | GG |
| S-11 | Male | 60 | 22.05 | 140/80 ,Yes | 50 | 10.19 | 15.77 |  | No | Yes | GT |
| S-12 | Female | 42 | 36.92 | 128/85,ys | 41 | 17.3 | 19 |  | No | No | GT |
| S-13 | Female | 40 | 25.81 | 120/80,yes | 39 | 18.47 | 27.43 |  | Yes | No | GT |
| S-14 | Female | 76 | 25.1 | 140/60, Yes | 68 | 10.54 | 13.42 |  | Yes | No | GT |
| S-16 | Female | 66 | 24.44 | 120/80,yes | 41 | 6.55 | 11.42 |  | Yes | No | GT |
| S-17 | Female | 50 | 23.94 | 160/90 , Yes | 30 | 7.22 | 9.67 |  | No | No | GT |
| S-18 | Male | 67 | 24 | 140/85 , Yes | 41 | 6 | 8 |  | Yes | No | GT |
| S-19 | Female | 57 | 24.14 | 150/100 ,Yes | 49 | 20.32 | 24.75 |  | No | No | GT |
| S-20 | Male | 60 | 21.72 | 140/90 ,Yes | 55 | 9.93 | 13.85 |  | Yes | No | GT |
| S-21 | Female | 40 | 24.44 | 110/60,yes | 38 | 7.7 | 12.63 |  | Yes | No | GT |
| S-22 | Male | 51 | 33.06 | 130/90, Yes | 43 | 14.72 | 22.36 |  | Yes | No | GG |
| S-23 | Female | 55 | 23.12 | 160/90 , Yes | 39 | 7.62 | 17.5 |  | Yes | No | GG |
| S-24 | Male | 63 | 19.03 | 150/60, Yes | 58 | 14.18 | 24.09 |  | Yes | No | GG |
| S-26 | Female | 46 | 23.31 | 140/90, Yes | 39 | 12.24 | 17.51 |  | **Yes, Stroke** | No | GT |
| S-27 | Male | 74 | 29.86 | 120/60, Yes | 50 |  |  | 9.74 | No | No,previously yes | GG |
| S-28 | Male | 64 | 26.39 | 160/60,Yes | 58 | 8.03 | 12.6 |  | **Yes, Stroke** | No | GG |
| S-29 | Female | 58 | 26.37 | 180/90, Yes | 47 | 6.36 | 8.57 |  | No | No | GT |
| S-31 | Male | 75 | 25.71 | 165/70, Yes | 48 |  |  | 8.58 | No | No | GG |
| S-32 | Male | 40 | 26.95 | 125/85 | 31 | 18.29 | 21.91 |  | **Yes, Stroke** | No | GT |
| S-33 | Female | 47 | 22.51 | 150/80, Yes | 32 | 6.9 | 9.7 |  | No | No | GT |
| S-35 | Female | 40 | 24.67 | 130/90,Yes | 32 | 20.03 | 27.02 |  | No | No | GG |
| S-36 | Female | 46 | 28.51 | 140/100, Yes | 30 | 15.25 | 23.53 |  | No | No | GG |
| S-37 | Male | 63 | 28.58 | 130/90, Yes | 40 |  |  | 4.74 | Yes | No,previously yes | GG |
| S-38 | Male | 70 | 19.15 | 125/85, Yes | 45 | 7.33 | 9.22 |  | No | No | GG |
| S-39 | Female | 65 | 28.57 | 140/95,Yes | 65 | 9.49 | 15.65 |  | No | No | GT |
| S-40 | Female | 59 | 25.39 | 140/85, Yes | 49 |  | 16.3 |  | No | No | GT |
| S-41 | Female | 52 | 30.3 | 140/90,Yes | 41 |  |  | 9.93 | No | No | GG |
| S-42 | Male | 65 | 24.45 | 140/90, Yes | 50 |  |  | 12.23 | **Yes, Stroke** | No | GG |
| S-43 | Female | 50 | 24.21 | 140/85, Yes | 43 | 8.59 | 14.95 |  | Yes | No | GG |
| **S-44** | Male | 64 | 23.03 | 150/90, Yes |  |  |  |  | **Brain Stroke** | No | GT |
| **S-45** | Female | 31 | 20.81 | 100/70,Yes | 27 |  |  | 16.46 | Yes | No | GG |
| **S-46** | Male | 60 | 30.43 | 150/90,Yes | 53 | 7.36 | 15.61 |  | Yes,Stroke | No | GG |
| **S-47** | Female | 55 | 25.54 | 140/90, Yes | 44 | 6.81 | 10.34 |  | No | No | GG |
| **S-49** | Female | 55 | 26.56 | 120/80, Yes | 40 | 8.56 | 17.78 |  | Yes(Pacemaker) | No | GG |
| **S-50** | Male | 46 | 20.99 | 140/90, Yes | 39 |  |  | 25.1 | No | No | GG |
| **S-51** | Female | 60 | 20.93 | 140/70, Yes | 45 |  |  | 13.65 | No | No | GT |
| **S-52** | Male | 55 | 22.1 | 110/65,yes | 54 | 13.4 | 21.76 |  | Yes,Stroke,Bypass | No | GG |
| **S-54** | Female | 31 | 32.05 | 130/80, Yes | 29 | 7.17 | 9.21 |  | Yes | No | GG |
| **S-56** | Male | 42 | 25.78 | 105/75, Yes | 38 | 5.09 | 15.59 |  | Yes | No | GT |
| **S-57** | Female | 54 | 28.4 | 150/85, Yes | 44 | 4.05 | 18.78 |  | No | No | GG |
| **S-59** | Female | 44 | 28.89 | 180/90,Yes | 38 | 7.86 | 11.77 |  | Yes | No | GT |
| **S-60** | Female | 82 | 22.48 | 140/80, Yes | 62 | 4.34 | 6.15 |  | **Brain Stroke** | No | GG |
| **S-61** | Female | 65 | 21.64 | 130/70, Yes | 38 |  | 11.76 |  | Open Heart Surgery | No | GG |
| **S-62** | Male | 71 | 19.43 | Low,110/70 | 42 |  |  | 9.82 | Yes, Block | No | GG |
| **S-63** | Male | 61 | 29.02 | 110/70,Yes | 56 |  |  | 7.66 | No | No | GT |
| **S-64** | Female | 55 | 22.89 | 110/70, Yes | 46 |  |  | 20.46 | Yes,Stroke | No | TT |
| **S-65** | Female | 70 | 16.23 | 110/70,Yes | 67 | 20.72 | 26.67 |  | Yes, Heart Attack | No | GG |
| **S-66** | Female | 43 | 25.39 | 140/80, Yes | 60 | 4.48 | 6.32 |  | Yes | No | GG |
| **S-67** | Female | 51 | 21.64 | 125/80, Yes | 27 |  |  | 14.21 | **Brain Stroke** | No | GG |
| **S-68** | Male | 65 | 26.57 | 135/90, Yes | 64 | 8.04 | 12.88 |  | No | No | **GT** |
| **S-69** | Female | 70 | 27.05 | 150/60, Yes | 55 |  |  | 11.43 | Yes | No | GG |
| **S-70** | Male | 48 | 23.87 | 160/90, Yes | 33 |  |  | 13.34 | **Brain Stroke** | No | GG |
| **S-71** | Female | 50 | 19.48 | 140/90, Yes | 36 | 16.27 | 20.45 |  | Yes | No | **GT** |
| **S-72** | Male | 68 | 25.15 | 150/80, Yes | 46 | 11.95 | 16.79 |  | No | No | GT |
| **S-73** | Female | 53 | 27.27 | 160/80, Yes | 38 | 13.46 | 15.95 |  | No | No | GT |
| **S-74** | Female | 50 | 28.12 | 140/85, Yes | 43 | 9.47 | 13.85 |  | No | No | GT |
| **S-75** | Female | 38 | 32.89 | 120/80, Yes | 28 | 8.81 | 10.84 |  | Yes, Stroke | No | GT |
| **S-76** | Female | 44 | 23.37 | 130/100, Yes | 36 | 9.49 | 11.17 |  | Yes, Stroke | No | TT |
| **S-77** | Female | 55 | 23.37 | 150/90, Yes | 55 | 8.2 | 12.27 |  | No | No | GG |
| **S-79** | Male | 51 | 20.58 | 120/80, Yes | 41 | 17.66 | 23.87 |  | **Brain Stroke** | Yes | GG |
| **S-80** | Male | 58 | 26.99 | 140/95, Yes | 54 | 6.34 | 11.35 |  | No | No | **GG** |
| **S-81** | Male | 62 | 25.71 | 150/80, Yes | 47 | 14.98 | 16.06 |  | No | No | GG |
| S-82 | Female | 49 | 20.81 | 140/90, Yes | 42 | 15.17 | 17.24 |  | No | No | **GG** |
| S-83 | Male | 65 | 28.37 | 160/90, Yes | 63 |  | 11.2 |  | Yes | No | GT |
| S-84 | Male | 52 | 25.34 | 100/75, Yes | 49 | 6.74 | 11.31 |  | Yes, Stroke, 2 Rings | Yes | **GG** |
| S-85 | Male | 65 | 21.45 | 120/80, Yes | 53 | 16.83 | 22.66 |  | Open Heart Surgery | No | **GG** |
| S-86 | Male | 57 | 27.28 | 160/80, Yes | 49 |  | 18,80 |  | No | No | **GG** |
| S-87 | Male | 62 | 27.05 | 130/90, Yes | 51 |  | 21.6 |  |  | No | **GG** |
| S-88 | Male | 62 | 25.51 | 160/40, Yes | 55 | 9.55 | 11.24 |  | No | No | **GT** |
| S-89 | Male | 49 | 26.4 | 140/90,Yes | 37 | 7.93 | 12.74 |  | No | No | **GT** |
| S-90 | Female | 42 | 25.97 | 140/80,Yes | 35 |  | 15.75 |  | No | No | **GT** |
| S-91 | Female | 45 | 19.98 | 180/60,Yes | 44 | 8.81 | 10.41 |  | No | No | **GT** |
| S-92 | Male | 62 | 22.39 | 130/80,Yes | 53 | 10.17 | 12.88 |  | Stroke, Heart Block | No | **GG** |
| S-93 | Female | 58 | 28.4 | 140/80,Yes | 49 | 16.31 | 19.43 |  | Yes | No | **TT** |
| S-94 | Female | 57 | 32.46 | 160/90,Yes | 56 | 10.19 | 12.58 |  | **Brain Stroke** | No | GG |
| S-95 | Female | 28 | 23.05 | 130/80,Yes | 25 | 10.15 | 20.55 |  | Yes | No | **GG** |
| S-97 | Female | 58 | 21.64 | 140/90,Yes | 50 | 11.84 | 20.41 |  | No | No | **GG** |
| S-98 | Female | 60 | 24.26 | 140/90,Yes | 58 | 8.73 | 9.48 |  | Yes | No | **GG** |
| S-101 | Female | 65 | 23.8 | 140/90 yes | 53 | 9.07 | 12.75 |  | yes | no | GT |
| S-102 | female | 60 | 27.18 | 140/85 yes | 52 |  | 16.2 |  | no | no | GT |
| S-106 | female | 62 | 28.13 | 140/85 yes | 57 |  | 17.78 |  | no | no | GT |
| S-108 | male | 67 | 23.29 | 140/70 yes | 65 | 10.41 | 14.16 |  | yes | no | GG |
| s-112 | Female | 49 | 23.7 | 140/80,Yes | 39 | 10.52 | 14.98 |  | no | no | GG |
| s-115 | Female | 48 | 22.6 | 140/90,Yes | 41 | 6.18 | 9.87 |  | no | no | GT |
| s-118 | Male | 63 | 22.8 | 130/80,Yes | 53 |  | 13.02 |  | yes block | no | GG |
| s-120 | Female | 46 | 28 | 140/90,Yes | 45 | 9.07 | 12.69 |  | no | no | GT |
| s-121 | Female | 48 | 29.2 | 140/90,Yes | 42 | 7.27 | 9.58 |  | yes heart failure | no | GG |
| s-123 | Female | 62 | 22.2 | 150/90,Yes | 52 | 16.91 | 23.74 |  | yes,heart weak | no | GG |
| S-124 | Female | 58 | 20.7 | 150/90,yes | 52 | 7.02 | 14.86 |  | No | No | GT |
| S-125 | Female | 76 | 23.3 | 140/80,yes | 72 | 9.87 | 17.33 |  | Yes | No | GG |
| S-126 | Male | 65 | 25.3 | 140/80,yes | 50 | 9.48 | 15.47 |  | Yes | No | GG |
| S-128 | Female | 35 | 29.1 | 115/70,yes | 33 | 8.97 | 14.01 |  | No | No | GG |
| S-129 | Female | 51 | 30.4 | 160/80,yes | 44 |  | 6.23 |  | No | No | GG |
| S-130 | Female | 53 | 37.1 | 140/90, yes | 49 |  | 9.56 |  | Yes | No | GG |
| S-131 | Female | 45 | 30.1 | 170/80, yes | 40 | 5.19 | 9.84 |  | No | No | GT |
| S-133 | Female | 39 | 28.7 | 160/80,yes | 34 |  | 13.75 |  | no | No | GG |
| S-134 | MALE | 49 |  |  |  |  |  |  |  |  | GG |
| s-135 | Male | 55 | 24.6 | 140/80,yes | 52 | 9.55 | 16.69 |  | yes,., ECG | yes | GT |
| s-136 | Female | 50 | 22.6 | 125/70,yes | 14 | 12.6 | 15.76 |  | yes | no | GG |
| s-137 | Female | 28 | 26.2 | 130/70,yes | 8months | 4.84 | 10.37 |  | no | no | TT |
| s-139 | Female | 55 | 22.9 | 140/80,yes | 44 | 9.64 | 17.3 |  | yes, | no | GG |
| s-141 | Male | 61 | 29.5 | 129\81,yes | 18 | 9.02 | 12.8 |  | yes | no | GG |
| s-142 | Female | 50 | 21.2 | 115/70,yes | 49 |  |  | 11.09 | no | no | GG |
| s-143 | Female | 55 |  | 120/65,yes |  |  |  | 11 | no | no | TT |
| s-144 | Female | 49 | 28.5 | 135/80,yes | 44 |  |  | 18.56 | no | no | GG |
| s-146 | Female | 32 | 18.9 | 140/90,yes | 25 | 15.54 | 24.95 |  | no | no | GG |
| s-148 | Male | 60 | 18.9 | 170/80,yes | 5 |  |  |  | no | no | GG |
| S-150 | Female | 69 |  | 160/70,med | 36 | 7.44 | 9.41 |  | Yes, ETT, Block | No | GG |
| S-151 | Male | 46 | 23.1 | 180/90,med | 38 | 8.43 | 12.64 |  | No | No | GT |
| S-152 | Female | 65 | 23.3 | 120/30,med,reg | 61 | 9.45 | 13.83 |  | No | No | GG |
| S-154 | Female | 51 | 22.5 | 120/80,med,reg | 31 |  |  | 5.81 | No | No | GG |
| S-155 | Female | 32 | 25 | 140/80,med | 30 | 5.9 | 7.98 |  | No | No | GG |
| S-156 | Female | 52 | 20.7 | 140/85,med | 45 |  |  | 14.41 | No, | No | GT |
| S-157 | Female | 60 | 22.4 | 120/80,med,reg | 48 | 6.29 | 11.37 |  | No | No | GG |
| S-158 | Female | 65 | 31.6 | 130/80,med,reg | 58 |  |  | 19.87 | No | No | GG |
| S-160 | Female | 52 | 23.7 | 130/90,med,reg | 35 |  |  | 8.91 | No | No | GT |
| S-161 | Female | 62 | 27.1 | 120/80,med,reg | 47 | 6.73 | 9.18 |  | No | No | GT |
| S-162 | Male | 68 | 94.6 | 140/60,med,reg | 50 | 7.62 | 12.38 |  | Yes, | No | GT |
| S-163 | Female | 65 | 25 | 110/70,med,reg | 50 |  |  | 11.07 | No | No | GG |
| S-164 | Female | 62 | 21.7 | 130/80,med,reg | 52 | 8.33 | 16.24 |  | No | No | GG |
| S-166 | Male | 61 | 28.8 | 130/80,med,reg | 51 | 12.32 | 17.57 |  | no | no | GG |
| S-168 | Female | 48 | 25 | 140/90,med,irregulr | 41 | 7.18 | 9.3 |  | no | no | TT |
| S-169 | Female | 50 | 26.2 | 130/80,med,reg | 40 |  |  | 15.49 | no | no | TT |
| S-170 | Female | 70 | 28.4 | 120/80,med,reg | 64 |  |  | 16.98 | no | no | GG |
| S-171 | Female | 60 | 21.5 | 120/80,med,reg |  | 7.8 | 14.39 |  | no | no | GT |
| S-172 | Female | 45 | 26.7 | 120/70,med,reg | 35 |  |  | 10.24 | no | no | GT |
| S-176 | Female | 39 | 23.7 | 180/110,med,irreglr | 31 | 6.6 | 12.81 |  | no | no | GG |
| S-177 | Female | 57 | 20.7 | 110/70,med,reg | 55 | 5.76 | 7.6 |  | no | no | GT |
| S-179 | Male | 55 | 22.4 | 120/80,med | 25 | 7.33 | 9.29 |  | no | no | GT |
| S-180 | Female | 58 | 27.7 | 130/80,yes,irregular | 43 | 6.63 | 9.47 |  | No | No | GG |
| S-181 | Female | 57 | 25.8 | 120/80,yes,regular | 42 | 4.9 | 10.46 |  | No | No | GG |
| S-182 | Female | 37 | 26.3 | 120/80,yes,regular | 33 | 11.39 | 15.17 |  | No | No | TT |
| S-183 | Male | 67 | 33.2 | 130/80,yes.regular | 47 | 10.37 | 12.88 |  | Yes,Block,Bypass | Yes | GG |
| S-185 | Male | 67 | 24.2 | 140/80,yes,regular | 61 | 6.99 | 8.15 |  | No | Yes | GG |
| S-189 | Female | 44 | 25.4 | 120/80,yes,regular | 33 |  |  | 6.5 | No | No | GG |
| S-191 | Male | 57 | 28.8 | 120/80,yes,regular | 49 |  | 10.95 |  | No | No | GG |
| S-192 | Male | 68 | 24.9 | 120/80,yes,regular | 38 | 7.95 | 13.15 |  | No | No | GG |
| S-193 | Female | 60 | 26.3 | 160/80,yes,regular | 57 |  |  | 5.35 | No | No | GT |
| S-200 | Female | 37 | 25 | 130/90,MED | 36 | 13.15 | 15.04 |  | No | No | TT |
| S-205 | Female | 55 | 27.6 | 130/40,Med,reg | 55 | 10.12 | 13.28 |  | Yes | No | GT |
| S-206 | Female | 69 | 21.4 | 120/80.med,reg | 56 | 9.48 | 20.71 |  | Yes | No | GG |
| S-207 | Female | 57 | 27.6 | 120/80,med,reg | 47 |  | 17.44 |  | No | No | GT |
| S-212 | Female | 42 | 30.8 | 120/80,med,reg | 41 | 6.13 | 8.19 |  | No | No | GG |
| S-213 | Female | 62 | 29.2 | 120/80,med,reg | 50 |  |  | 8.03 | Yes | No | GG |
| S-214 | Female | 50 | 23.3 | 120/80.med,reg | 48 | 9.94 | 15.21 |  | No | No | GT |
|  |  |  |  |  |  |  |  |  |  |  |  |
|  |  |  |  |  |  |  |  |  |  |  |  |
